# Establishment of the *Stenothemus volaticus* group (Coleoptera, Cantharidae), with description of two new species from Xizang, China

**DOI:** 10.3897/zookeys.1289.192623

**Published:** 2026-08-14

**Authors:** Ruobing Hua, Shujuan Ge, Haoyu Liu, Xingke Yang, Yuxia Yang

**Affiliations:** 1 Key Laboratory of Zoological Systematics and Application, College of Life Sciences, Hebei University, Baoding 071002, Hebei Province, China Key Laboratory of Zoological Systematics and Application, College of Life Sciences, Hebei University Baoding China; 2 Hebei Basic Science Center for Biotic Interaction, Hebei University, Baoding 071002, China Hebei Basic Science Center for Biotic Interaction, Hebei University Baoding China; 3 Key Laboratory of Zoological Systematics and Evolution, Institute of Zoology, Chinese Academy of Sciences, Beijing 100101, China Key Laboratory of Zoological Systematics and Evolution, Institute of Zoology, Chinese Academy of Sciences Beijing China

**Keywords:** Alpha taxonomy, Himalayas, identification key, soldier beetles, SW China

## Abstract

The *Stenothemus volaticus* species group is proposed, comprising a total of 13 species from the Himalayas and southwestern China. Within this group, two new species are described under the names of *S. dromoensis***sp. nov**., and *S. liangi***sp. nov**. Meanwhile, the female internal reproductive organs of *S. schneideri* Švihla, 2004 and *S. volaticus* Champion, 1925 are illustrated for the first time. An identification key for the species and a distribution map of the *S. volaticus* species group are provided.

## Introduction

Soldier beetles (family Cantharidae Imhoff, 1856) represent one of the major lineages within the superfamily Elateroidea Leach, 1815, comprising over 6600 species worldwide and ranking second in diversity only to click beetles (Kundrata 2026). The family exhibits particularly high diversity in the Oriental and eastern Palaearctic regions, where many cantharid lineages originated and to which many remain restricted, as exemplified in the genus *Lycocerus* Gorham, 1889 *sensu lato* (Okushima 2005). This pattern is also exhibited in *Stenothemus* Bourgeosis, 1907.

Since Wittmer’s (1974) revision, the genus *Stenothemus* has become increasingly diverse, with dozens of new species discovered in recent years (Okushima and Satô 1997, 1999; Švihla 2004, 2005, 2011; Yang et al. 2014, 2021a, 2021b; Hsiao 2015; Hsiao et al. 2016; Ge et al. 2021a, 2021b, 2021c). Currently, it contains approximately 100 species widely distributed across the East Palaearctic and Indochina (Ge et al. 2021a, 2021b, 2021c; Yang et al. 2021a, 2021b). To facilitate taxonomic organization, a species-group classification was first introduced by Švihla (2005) to this genus and subsequently adopted by Yang et al. (2014) and Ge et al. (2021b, 2021c). To date, three species groups have been proposed, including the *S. harmandi* group (Švihla 2005), the *S. notaticollis* group (Ge et al. 2021c), and the *S. laterophysus* group (Ge et al. 2021c). These groups are primarily distributed in the Himalayas, with a few species extending into southwest China, a region recognized as a hotspot of cantharid diversity (Liu et al. 2022). Here, we recognize an additional group from this area, which includes *S. volaticus* Champion, 1925 and its sibling species (Wittmer 1974; Švihla 2004; Yang et al. 2021b), as well as two new species discovered from Xizang, China.

## Material and methods

The studied specimens are deposited in the following collections:

**IZAS** Institute of Zoology, Chinese Academy of Sciences, Beijing, China;

**NHMB** Naturhistorisches Museum Basel, Basel, Switzerland;

**NMPC** National Museum, Prague, Czech Republic.

The genitalia of both sexes, along with the female abdominal sternites VIII, were dissected and cleared in 10% NaOH solution. Male genitalia were subsequently immersed in a warm 30% NaOH solution for an appropriate duration (generally 5 to 15 minutes, depending on their size and condition), after which they were cleaned. Female internal genitalia were stained with haematoxylin. Habitus photos were taken using a Canon EOS 80D digital camera, while images of the genitalia were captured with a Leica M205A stereomicroscope. All images were processed using Helicon Focus 7 and edited in Adobe Photoshop CS5. Body length was measured from the anterior margin of the clypeus to the elytral apex, and body width was assessed across the humeral part of the elytra.

For type specimens of the previously known species, complete label data were provided. Square brackets “[]” were used to indicate remarks or comments, [p] denotes printed data, and [h] handwritten data. Quotation marks were used to separate data from different labels, and a backslash “/” to separate data from different lines on the same label. For additional specimens, quotation marks were used when the original labels were written in English, while labels originally in Chinese were transliterated into English.

## Taxonomy

### Class Insecta Linnaeus, 1758

**Order Coleoptera Linnaeus, 1758**

**Superfamily Elateroidea Leach, 1815**

**Family Cantharidae Imhoff, 1856**

**Subfamily Cantharinae Imhoff, 1856**

**Genus *Stenothemus* Bourgeois, 1907**

### *Stenothemus volaticus* species group

**Diagnosis**. Body (Figs 2, 5; Yang et al. 2021b: figs 1A, B, E, 5A, 9D) small to middle-sized (5.0–11.2 mm in length), black, blackish or yellowish brown, elytra usually paler at humeri. Eyes strongly protruding, vertex behind eyes strongly narrowed posteriorly, head width across eyes almost as wide as pronotum. Pronotum nearly as long as wide, posterior angles acute and feebly projecting laterally. Elytra elongate, at least 5.5 times longer than pronotum. Aedeagus ovate, with dorsal plate of each paramere narrowed apically (Fig. 6; Wittmer 1974: figs 9, 10, 16; Švihla 2004: figs 148, 152, 154, 156, 160, 163, 164; Yang et al. 2021b: fig. 10H). Female internal reproductive organ: vagina abruptly thinned into a ventro-apical tube, where diverticulum and spermathecal duct arise, spermatheca extending into a tube at base, where accessory gland opening, diverticulum, spermatheca and accessory gland at most moderately long (Fig. 3; Yang et al. 2021b: figs 4A, D, 8B).

**Figure 1.**
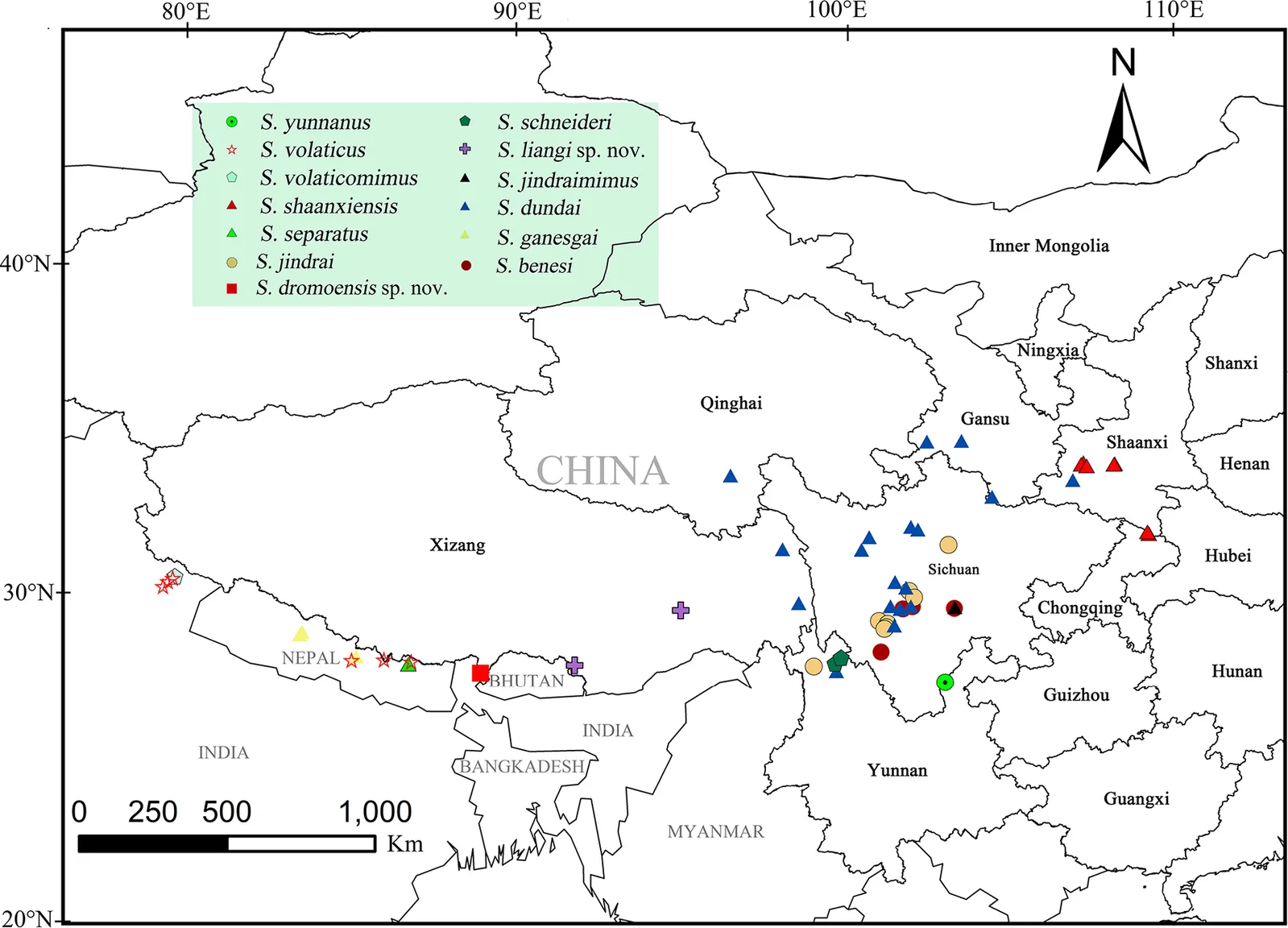
Distribution map of the *Stenothemus volaticus* species group.

**Figure 2.**
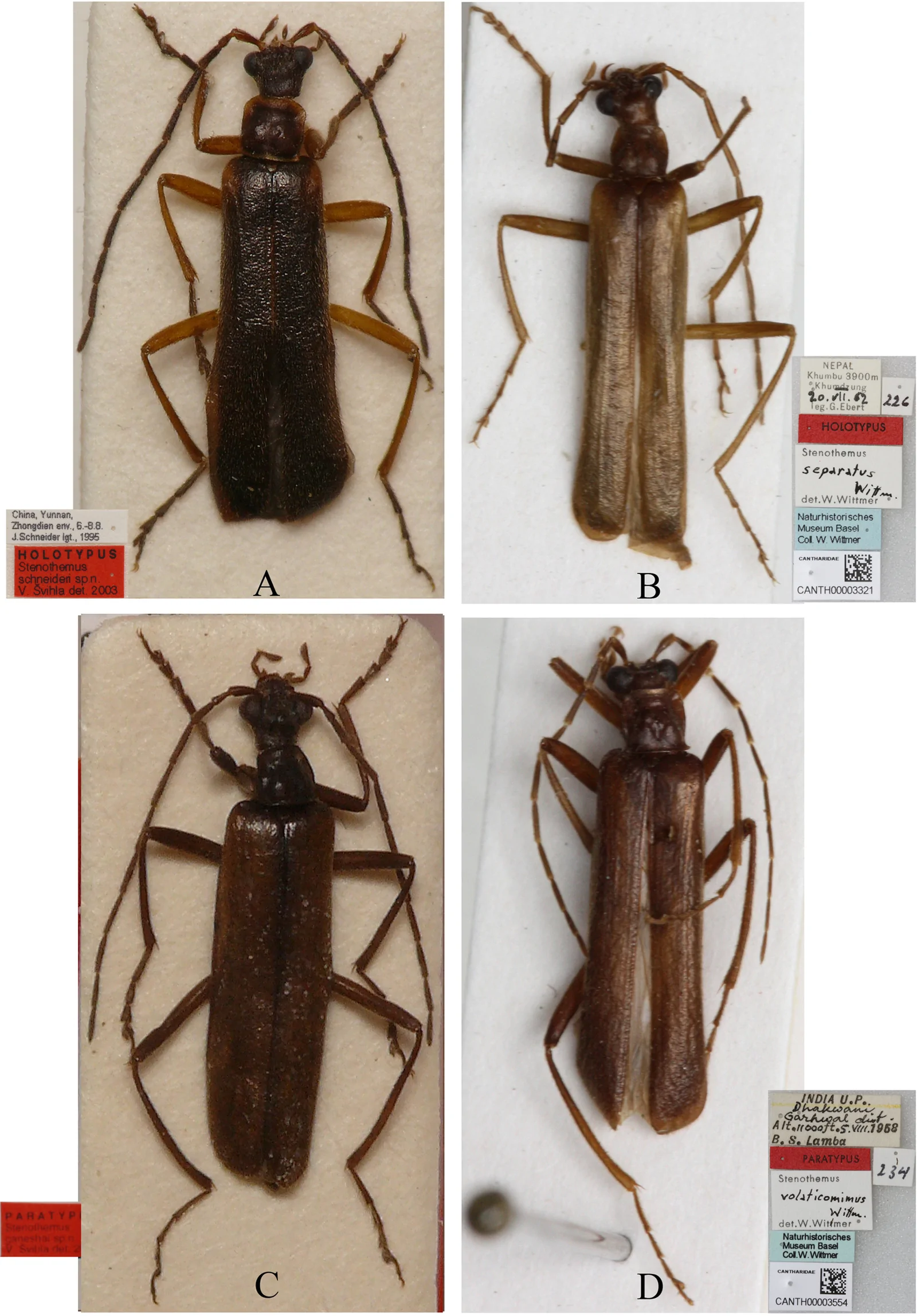
Male habitus, dorsal view. **A**. *Stenothemus schneideri* Švihla, 2004; **B**. *S. separatus* Wittmer, 1974; **C**. *S. ganeshai* Švihla, 2004; **D**. *S. volaticomimus* Wittmer, 1974.

**Figure 3.**
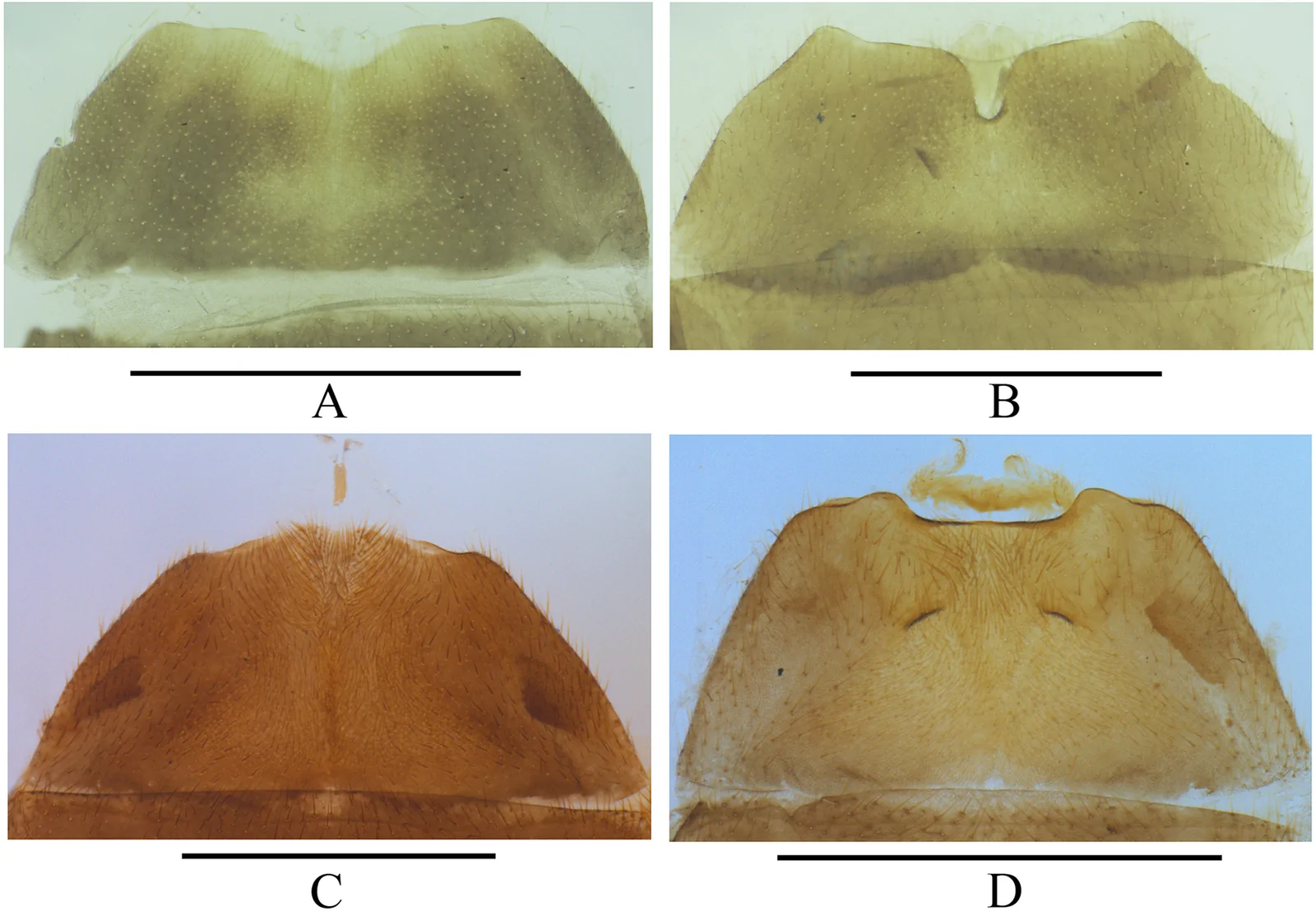
Female abdominal sternite VIII, ventral view. **A**. *Stenothemus schneideri* Švihla, 2004; **B**. *S. volaticus* Champion, 1925; **C**. *S. dromoensis* sp. nov.; **D**. *S. liangi* sp. nov. Scale bars: 1.0 mm.

**Included species**. *Stenothemus benesi* Švihla, 2004, *S. dundai* Švihla, 2004, *S. schneideri* Švihla, 2004, *S. jindrai* Švihla, 2004, *S. jindraimimus* Y. Yang & X. Yang, 2021, *S. shaanxiensis* Švihla, 2004, *S. ganeshai* Švihla, 2004, *S. separatus* Wittmer, 1974, *S. volaticomimus* Wittmer, 1974, *S. volaticus* Champion, 1925, *S. yunnanus* Švihla, 2004, *S. dromoensis* sp. nov., and *S. liangi* sp. nov.

**Distribution**. (Fig. 1) SW China, India, Nepal.

**Remarks**. This species group can be easily differentiated from other *Stenothemus* species by the blackish or yellowish brown elytra, usually paler at humeri (except *S. dundai*), not uniformly black or light brown with numerous irregular dark brown markings; elongate elytra, at least 5.5 times longer than pronotum, instead of stout and less than 4.0 times; aedeagus generally ovate as a whole, not greatly shrunked in middle or apically (in the *S. harmandi*, *S. laterophysus* and *S. notaticollis* species groups, Ge et al. 2021b: figs 2, 3; 2021c: fig. 3); ventral process and dorsal plate of each paramere simple, not bent ventrally or expaned apically (in the *S. harmandi* species group, Ge et al. 2021b: figs 2, 3); female abdominal tergite VIII normal, not curled ventrally on both sides (in the *S. harmandi* species group, Ge Yang et al. 2014: figs 9, 10); vagina abruptly thinned into a relatively short and thin ventro-apical tube, not long and stout (in the *S. notaticollis* species group, Ge et al. 2021c: fig. 4C); diverticulum and spermathecal at most moderately long, instead of extremely long (in the *S. laterophysus* species group, Ge et al. 2021c: fig. 4A).

### Key to the species of the *Stenothemus volaticus* species group

**Table:** 

1	Body uniformly black, rarely paler on posterior angles of pronotum or elytral humeri (Yang et al. 2021b: fig. 1E); aedeagus: dorsal plate of each paramere curled ventrally at inner-apical angles, laterophyses separated on both sides of median lobe, with apices acute and directing inner-dorsally (Švihla 2004: fig. 154)	***S. dundai* Švihla, 2004**
–	Body bicolored, dark or light brown, or yellow, or mixed with black; aedeagus unlike above	**2**
2	Elytra black, yellowish brown only at humeral bulges (Fig. 2A); aedeagus: dorsal plate of each paramere with a rectangular protuberance at inner margin, middle emargination between them shallower, no more than 1/3 of the whole length (Švihla 2004: fig. 160)	***S. schneideri* Švihla, 2004**
–	Elytra dark or light brown, or paler at least basal 1/3 parts extending from humeri (Fig. 2B–D, 5; Yang et al. 2021b: figs 1A, B, 5A, 9D); aedeagus: dorsal plate of each paramere without any protuberance at inner margin, middle emargination between them deeper, at least half of the whole length (Fig. 6; Wittmer 1974: figs 9, 10, 16; Švihla 2004: figs 148, 152, 156, 163, 164; Yang et al. 2021b: fig. 10H)	**3**
3	Aedeagus: dorsal plate of each paramere abruptly narrowed apically, acute at apex and directed to each other (Švihla 2004: fig. 152, 156; Yang et al. 2021b: fig. 10H)	**4**
–	Aedeagus: dorsal plate of each paramere progressively narrowed apically, rounded or truncate apex (Fig. 6; Wittmer 1974: figs 9, 10, 16; Švihla 2004: figs 148, 163, 164)	**6**
4	Elytra uniformly yellowish brown (Fig. 5B); aedeagus: dorsal plate of each paramere strongly narrowed apically, arcuate at inner margin, not hook-like at apices (Švihla 2004: fig. 152)	***S. yunnanus* Švihla, 2004**
–	Elytra bicolored or yellow; aedeagus: dorsal plate of each paramere moderately narrowed apically, sinuate at inner margin, hook-like at apices	**5**
5	Body smaller (6.6 mm in length); elytra uniformly light brown (Yang et al. 2021b: fig. 9D); aedeagus: middle emargination between dorsal plates shallower and nearly as long as half of the whole length (Yang et al. 2021b: fig. 10H)	***S. jindraimimus* Y. Yang & X. Yang, 2021**
–	Body larger (7.5–10.0 mm in length); elytra dark brown, yellowish brown on humeri (Yang et al. 2021b: fig. 5A); aedeagus: middle emargination between dorsal plates deeper and much longer than half of the whole length (Švihla 2004: fig. 156)	***S. jindrai* Švihla, 2004**
6	Aedeagus: laterophyses closely next to each other, with only a slit separating them, rounded at apices (Fig. 6B; Wittmer 1974: fig. 10)	**7**
–	Aedeagus: laterophyses clearly separated at apical parts (Wittmer 1974: fig. 9)	**8**
7	Aedeagus: laterophyses papillary at apices (Wittmer 1974: fig. 10)	***S. volaticomimus* Wittmer, 1974**
–	Aedeagus: laterophyses widely rounded at apices (Fig. 6B)	***S. dromoensis* sp. nov**.
8	Aedeagus: laterophyses widely separated on both sides of median lobe, with apices directing laterally or apically (Wittmer 1974: fig. 16; Švihla 2004: fig. 148)	**9**
–	Aedeagus: laterophyses positioned on dorsal side of median lobe, with apices facing or directing to each other	**10**
9	Aedeagus: laterophyses beak-like at apices and directing laterally, without keel on ventral side (Švihla 2004: fig. 148)	***S. ganeshai* Švihla, 2004**
–	Aedeagus: laterophyses with apices tapered and directing apically, each with a sharp longitudinal keel on ventral side (Wittmer 1974: fig. 16)	***S. separatus* Wittmer, 1974**
10	Aedeagus: laterophyses generally hook-like, with apices rounded and facing to each other (Fig. 6E; Wittmer 1974: fig. 9)	**11**
–	Aedeagus: laterophyses not hook-like, with apices acute and directed to each other (Švihla 2004: figs 163, 164)	**12**
11	Aedeagus: dorsal plates of parameres truncate at apical margins, with inner margins diverging apically (Wittmer 1974: fig. 9)	***S. volaticus* Champion, 1925**
–	Aedeagus: dorsal plates of parameres narrowly rounded at apical margin, with inner margins converging apically (Fig. 6F)	***S. liangi* sp. nov**.
12	Body larger (8.2–10.8 mm in length), dark brown, paler on elytral humeri; aedeagus: dorsal plate of each paramere simple on inner surface (Švihla 2004: fig. 163)	***S. benesi* Švihla, 2004**
–	Body smaller (5.5–8.3 mm in length), uniformly yellow (Yang et al. 2021b: fig. 1B); aedeagus: dorsal plate of each paramere with a lateroapical keel on inner surface (Švihla 2004: fig. 164)	***S. shaanxiensis* Švihla, 2004**

#### Stenothemus benesi

Taxon classificationAnimaliaColeopteraCantharidae

Švihla, 2004

1FE969F2-793B-5377-9569-C09F12A5CDAE

Fig. 1

Stenothemus benesi Švihla, 2004: 202, figs 161–163, 214; Yang et al. 2021b: 3, fig. 1A.

##### Distribution.

(Fig. 1) China (Sichuan, Yunnan).

#### Stenothemus dundai

Taxon classificationAnimaliaColeopteraCantharidae

Švihla, 2004

697CCB5F-ED14-5CB5-A793-F634A2E4DC0E

Fig. 1

Stenothemus dundai Švihla, 2004: 199, figs 153–154, 212; Yang et al. 2021b: 11, figs 1E, 3D, 4D.

##### Distribution.

(Fig. 1) China (Gansu, Qinghai, Shaanxi, Sichuan, Yunnan, Xizang).

#### Stenothemus jindrai

Taxon classificationAnimaliaColeopteraCantharidae

Švihla, 2004

FDEF31A4-CD1D-56CA-AF75-7B635D729CF0

Fig. 1

Stenothemus jindrai Švihla, 2004: 200, figs 157–158; Yang et al. 2021b: 13, figs 3F, 5A, 8B.

##### Distribution.

(Fig. 1) China (Sichuan, Yunnan).

#### Stenothemus jindraimimus

Taxon classificationAnimaliaColeopteraCantharidae

Y. Yang & X. Yang, 2021

91F9F2AA-FCC7-5D57-A223-9B9FFDFCE349

Fig. 1

Stenothemus jindraimimus Y. Yang & X. Yang, in: Yang et al. 2021b: 28, figs 9D, 10G–I.

##### Distribution.

(Fig. 1) China (Sichuan).

#### Stenothemus shaanxiensis

Taxon classificationAnimaliaColeopteraCantharidae

Švihla, 2004

D11A947A-42F0-5EDE-9D69-77AE46BF6CA0

Fig. 1

Stenothemus benesi shaanxiensis Švihla, 2004: 202, fig. 164.
Stenothemus shaanxiensis : Yang et al. 2021b: 3, figs 1B, 2A–C, 3A, 4A.

##### Distribution.

(Fig. 1) China (Shaanxi, Chongqing).

#### Stenothemus schneideri

Taxon classificationAnimaliaColeopteraCantharidae

Švihla, 2004

A4D74DA8-20C8-5BA3-8AEE-320AFD459551

Figs 1, 2A, 3A, 4A

Stenothemus schneideri Švihla, 2004: 201, figs 159–160.

##### Type material examined.

***Holotype***: 1♂ (NMPC), [p] “China, Yunnan, / Zhongdien env., 6.-8.-8. / J. Schneider lgt., 1995”, [p] “HOLOTYPUS / Stenothemus / schneideri sp. n. / V. Švihla det. 2003”.

**Figure 4.**
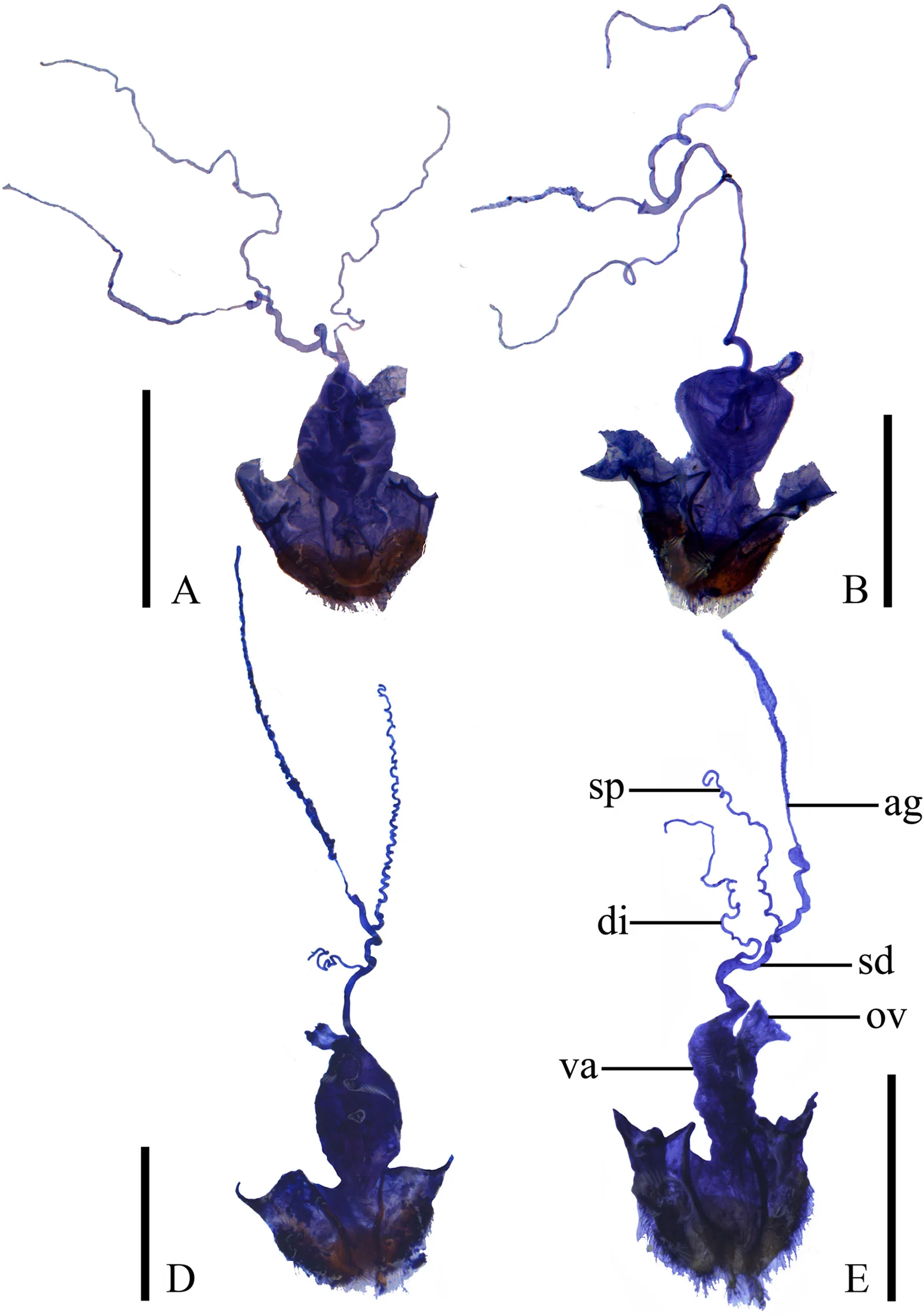
Female internal reproductive organ, lateral view. **A**. *Stenothemus schneideri* Švihla, 2004; **B**. *S. volaticus* Champion, 1925; **C**. *S. dromoensis* sp. nov.; **D**. *S. liangi* sp. nov. Scale bars: 1.0 mm. Abbreviations: ag = accessory gland, di = diverticulum, ov = median oviduct, sd = spermathecal duct, sp = spermatheca, va = vagina.

##### Additional material examined.

China • Yunnan: 2♀♀ (IZAS), Zhongdian [Xianggelila], Geza, 3200 m, 1981.VIII.8, leg. Shuyong Wang.

##### Descriptive notes.

Female. Abdominal sternite VIII (Fig. 2A) widely and triangularly emarginate in middle of posterior margin, behind the middle emargination with a membranous lobe. Internal reproductive organ (Fig. 3A): diverticulum moderately long, spermathecal duct thinner and longer than the ventro-apical tube of vagina, spermatheca as long as diverticulum, accessory gland evenly thin along whole length, almost as long as spermatheca, the basal tube of spermatheca as long as 1/4 length of spermathecal duct.

##### Distribution.

(Fig. 1) China (Yunnan).

##### Remarks.

The male habitus (Fig. 2A) is provided for the first time.

#### Stenothemus separatus

Taxon classificationAnimaliaColeopteraCantharidae

Wittmer, 1974

AEAE8C22-487F-566F-A357-6803DCA38ACA

Figs 1, 2B

Stenothemus separatus Wittmer, 1974: 58, fig. 16.

##### Type material examined.

***Holotype***: 1♂ (NHMB), [p] “NEPAL / Khumbu 3900m / Khumdzung / 20.VII.(19)62 / leg. G. Ebert”, [p] “HOLOTYPUS”, [p-h] “Stenothemus / separatus / Wittm. / det. W. Wittmer”, [p] “Naturhistorisches / Museum Basel / Coll. W. Wittmer”, [p] “CANTHARIDAE / CANTH00003321”.

##### Distribution.

(Fig. 1) Nepal.

##### Remarks.

The male habitus (Fig. 2B) is provided for the first time to make comparison with others.

#### Stenothemus ganeshai

Taxon classificationAnimaliaColeopteraCantharidae

Švihla, 2004

FBEB0020-BF01-57BC-AD3F-8A6DEFE21CDF

Figs 1, 2C

Stenothemus ganeshai Švihla, 2004: 197, figs 148, 211.

##### Type material examined.

***Paratype***: 1♂ (NMPC), [p] “Nepal, Myagdi distr. / S-slope Ruyachar / Duri, 32-3300m, 23.vi. / 1998 BERNDT / SCHMIDT”, [p] “PARATYPUS / Stenothemus / ganeshai sp. n. / V. Švihla det. 2003”.

##### Distribution.

(Fig. 1) Nepal.

##### Remarks.

The male habitus (Fig. 2C) is provided for the first time to make comparison with others.

#### Stenothemus volaticomimus

Taxon classificationAnimaliaColeopteraCantharidae

Wittmer, 1974

936CCAB5-D252-56C4-BF51-2E263D363BC5

Figs 1, 2D

Stenothemus volaticomimus Wittmer, 1974: 54, fig. 10.

##### Type material examined.

***Paratype***: 1♂ (NHMB), [p] “INDIA U.P. / Dhakwani / Garhwal Distr. / Alt. 11000ft. 5.VIII.1958 / B. S. Lamba”, [p] “PARATYPUS”, [p-h] “Stenothemus / volaticomimus / Wittm. / det. W. Wittmer”, [p] “Naturhistorisches / Museum Basel / Coll. W. Wittmer”, [p] “CANTHARIDAE / CANTH00003554”.

##### Distribution.

(Fig. 1) India.

##### Remarks.

The male habitus (Fig. 2D) is provided for the first time to make comparison with others.

#### Stenothemus volaticus

Taxon classificationAnimaliaColeopteraCantharidae

Champion, 1925

D668BBCB-98D8-5534-8930-2B0EA5BD19EF

Figs 1, 3B, 4B, 5A

Stenothemus volaticus Champion, 1925: 265; Wittmer 1974: 54; fig. 9; Yang and Yang 2013: 19.

##### Determined material examined.

1♂ (NHMB), [p] “Burphu, / Gori V., 11500 ft. / India. H. G. C.”, [p] “G. C. Champion / Brit. Mus. / 1925—42.”, [p] “Stenothemus volaticus Champ.”, [p] “Naturhistorisches / Museum Basel / Coll. W. Wittmer”.

**Figure 5.**
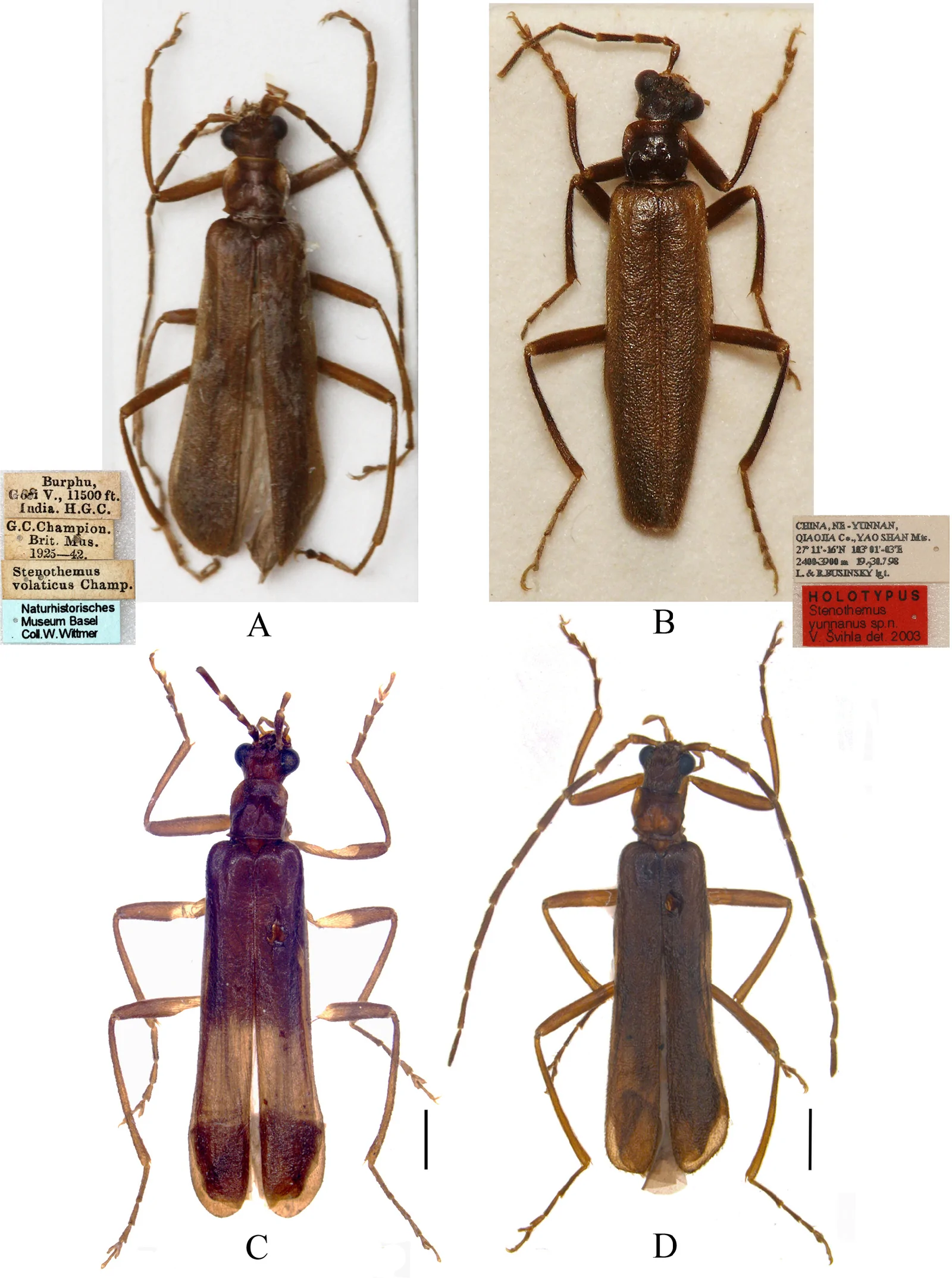
Male habitus, dorsal view. **A**. *Stenothemus volaticus* Champion, 1925; **B**. *S. yunnanus* Švihla, 2004; **C**. *S. dromoensis* sp. nov.; **D**. *S. liangi* sp. nov. Scale bars: 1.0 cm.

##### Additional material examined.

1♂ (IZAS), China • Xizang, Zham, Youxiang, 3400 m, 1975.VII.7, leg. Fusheng Huang; • 1♂ (IZAS), same locality as the preceding, 1975.VII.6, leg. Xuezhong Zhang; • 1♀ (IZAS), same locality as the preceding, 3300 m, 1975.VII.6, leg. Fusheng Huang.

##### Descriptive notes.

Abdominal sternite VIII (Fig. 3B) narrowly and roundly emarginate in the middle of the posterior margin, behind the middle emargination with a membranous lobe.

Internal reproductive organ (Fig. 4B): diverticulum moderately long, spermathecal duct as long as half of the ventro-apical tube of vagina, spermatheca as long as diverticulum, accessory gland evenly thin along whole length, almost as long as spermatheca, the basal tube of spermatheca very short, as long as 1/4 length of spermathecal duct.

##### Distribution.

(Fig. 1) China (Xizang), India, Nepal.

##### Remarks.

The male habitus (Fig. 5A) is provided for the first time to make comparison with others.

#### Stenothemus yunnanus

Taxon classificationAnimaliaColeopteraCantharidae

Švihla, 2004

90A3C512-B0C2-5543-8479-C57894E2D23A

Figs 1, 5B

Stenothemus yunnanus Švihla, 2004: 198, figs 151–152.

##### Type material examined.

***Holotype***: 1♂ (NMPC), [p] “CHINA, NE-YUNNAN, / QIAOJIA Co., YAO SHAN Mts. / 27°11'-16'N 103°01'-03' / 2400-3900m 19-30.7.(19)98 / L. & R. BUSINSKY lgt.”.

##### Distribution.

(Fig. 1) China (Yunnan).

##### Remarks.

The male habitus (Fig. 5B) is provided for the first time to make comparison with others.

#### Stenothemus dromoensis

Taxon classificationAnimaliaColeopteraCantharidae

Y. Yang, Ge & X. Yang sp. nov.

0EBAB5E6-1660-5D96-A35D-2C03CB0AE3E2

https://zoobank.org/6E462BA2-7492-4BD4-A9AD-E7E8C1CFB7B6

**Figure 6.**
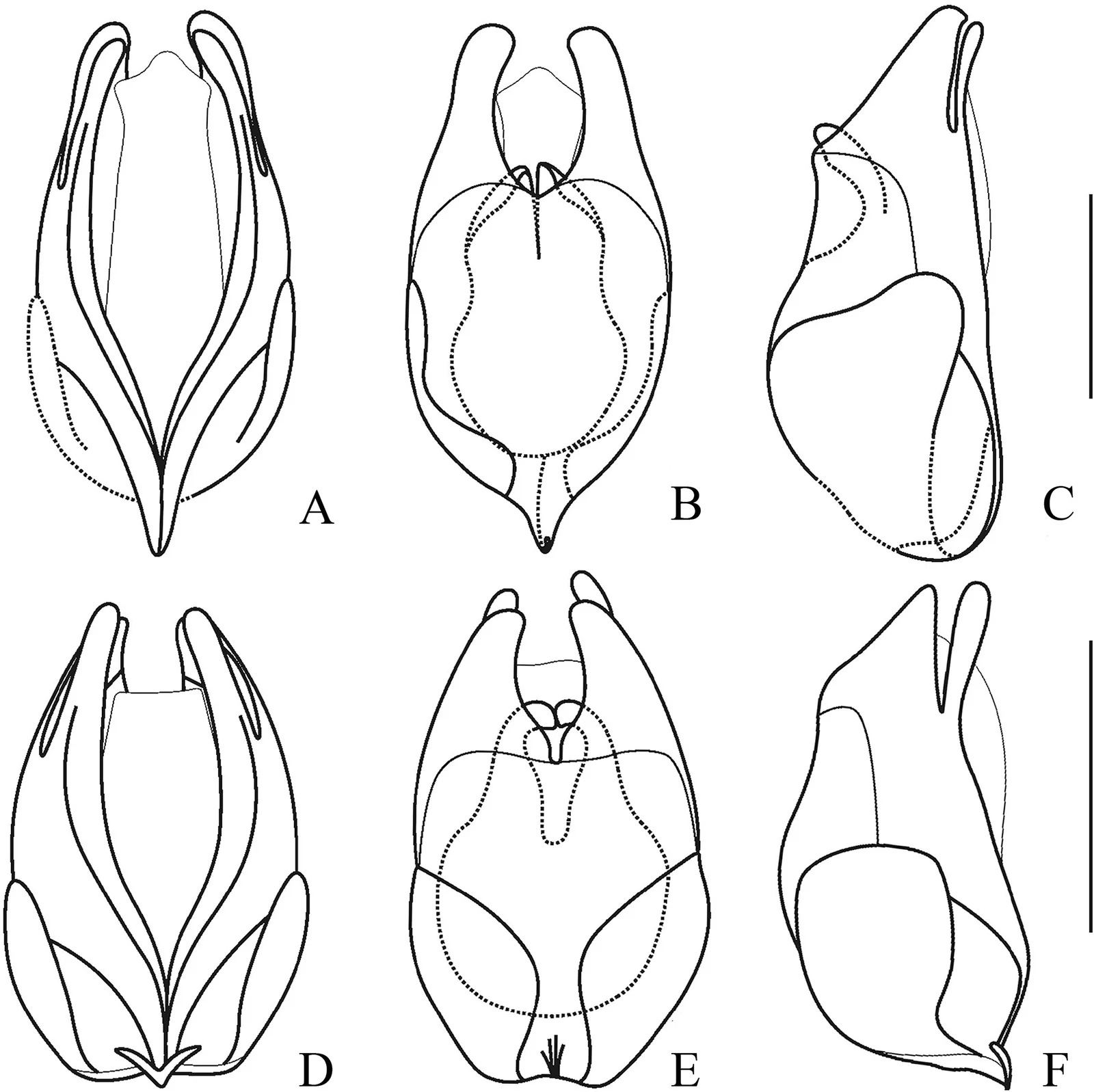
Aedeagus (**A, D**. ventral view; **B, E**. dorsal view; **C, F**. lateral view). **A–C**. *Stenothemus dromoensis* sp. nov.; **D–F**. *S. liangi* sp. nov. Scale bars: 0.5 mm.

Figs 1, 3C, 4C, 5C, 6A–C

##### Type material.

***Holotype***: • ♂ (IZAS), China, Xizang, Dromo, 2800 m, 1976.VIII.19, leg. Yiran Zhang. ***Paratype***: • 1 ♀ (IZAS), same data as the holotype.

##### Description.

***Body*** 8.5–11.2 mm in length (8.5 mm in the holotype), 1.5–2.5 mm in width (1.5 mm in the holotype).

**Male**. (Fig. 5C) Head reddish brown, darkened at clypeus, pronotum dark brown, reddish brown at anterior angles, scutellum reddish brown, elytra dark brown, legs yellow at bases of femora.

***Head*** subquadrate, vertex strongly narrowed posteriorly, with a pair of smooth rectangular impressions behind antennal sockets; eyes strongly protruding, head width across eyes as wide as pronotum; antennae filiform, antennomeres III about 1.5 times as long as II (IV – XI missing).

***Pronotum*** subrounded, as long as wide, anterior margin arcuate, lateral margins obliquely converging posteriorly and confluent with anterior angles, posterior margin nearly straight, posterior angles acute and projecting laterally, disc distinctly convex on postero-lateral parts, surface finely and densely punctate.

***Elytra*** elongate, feebly widened posteriorly, 4.0 times as long as wide, 6.4 times as long as pronotum, surface densely and finely punctate, longitudinal costae hardly visible.

***Aedeagus*** (Fig. 6A – C): dorsal plate of each paramere nearly as long as ventral process (Fig. 6C), progressively narrowed apically, rounded at apex and acute at inner-apical angle, arcuate at inner margin (Fig. 6B); laterophyses closely next to each other at apical parts, with only a slit separating them, rounded at apices (Fig. 6B).

**Female**. Similar to male, but body larger, eyes smaller, pronotum wider and 1.2 times as wide as long, elytra with lateral margins moderately diverging posteriorly. Abdominal sternite VIII (Fig. 3C) shallowly and roundly emarginate on both sides and in middle of posterior margin, the middle emargination shallower and narrower than lateral ones, behind it with a membranous lobe. Internal reproductive organ (Fig. 4C): diverticulum thin and short, spermathecal duct nearly as long as ventro-apical tube of vagina, spermathecal about 3.0 times as long as diverticulum, accessory gland evenly thin and almost as long as spermatheca, the tube at base of spermatheca shorter than spermathecal duct.

##### Diagnosis.

The new species is similar to *S. volaticomimus* in the aedeagus: laterophyses closely next to each other, with only a slit separating them, but can be differentiated from the latter by the combination of following characters: head and scutellum reddish brown, elytra almost black, legs bicolored (Fig. 5C), while uniformly dark brown in the latter (Fig. 2D); pronotum subrounded, with lateral margins obliquely converging posteriorly, while subquadrate, with lateral margins nearly parallel in the latter (Fig. 2D); aedeagus: laterophyses widely rounded at apices (Fig. 6B), while papillary in the latter (Wittmer 1974: fig. 10).

##### Etymology.

The specific name is derived from the type locality, Dromo, Xizang Autonomous Region, China.

##### Distribution.

(Fig. 1) China (Xizang).

#### Stenothemus liangi

Taxon classificationAnimaliaColeopteraCantharidae

Y. Yang, Ge & X. Yang sp. nov.

6B296825-1A44-536E-A2D9-DB3F4182D244

https://zoobank.org/25111DE7-0878-4B59-9910-DC932B1B1B31

Figs 1, 3D, 4D, 5D, 6D–F

##### Type material.

***Holotype***: • ♂ (IZAS), China, Xizang, Cona, Lexiang, Frontier Defense Highway of the 6^th^ Company 17 km, 27.7950°N, 91.7734°E, 3691 m, 2016.VI.5, leg. Hongbin Liang & Qingtao Wu. ***Paratype***: • 1 ♀ (IZAS), China, Xizang, Beibung, Lage, 29.4672°N, 95.0011°E, 3220 m, 2016.VIII.8, leg. Hongbin Liang.

##### Description.

***Body*** 7.5–8.6 mm in length (7.5 mm in the holotype), 1.3–1.7 mm in width (1.3 mm in the holotype).

**Male**. (Fig. 5D) Body brown, elytra blackish brown.

***Head*** subquadrate, vertex strongly narrowed posteriorly, with a pair of smooth rectangular impressions behind antennal sockets; eyes strongly protruding, head width across eyes as wide as pronotum; antennae filiform, extending to apical 1/4 length of elytra, antennomeres III about 1.5 times as long as II, IV – XI each with a smooth oblong impression in middle of outer margin, XI feebly longer than X, pointed at apices.

***Pronotum*** subquadrate, about 1.1 times as long as wide, anterior margin nearly straight, lateral margins sinuate, posterior margin nearly straight, anterior angles subrounded, posterior angles acute and projecting laterally, surface sparsely and finely punctate, disc distinctly convex on postero-lateral parts.

***Elytra*** elongate, about 4.1 times as long as humeral width, 6.8 times as long as pronotum, disc densely and finely punctate, longitudinal costae hardly visible.

***Aedeagus*** (Fig. 6D – F): dorsal plate of each paramere as long as ventral process (Fig. 6F), progressively narrowed apically, rounded at apex, sinuate at inner margin (Fig. 6D); laterophyses separated at apical parts, generally hook-like, with apices rounded and facing each other (Fig. 6E).

**Female**. Similar to male, but body larger, eyes smaller, antennae shorter and extending to half-length of elytra, antennomeres IV – XI without any impressions, elytra about 3.6 times as long as humeral width. Abdominal sternite VIII (Fig. 3D) shallowly and rectangularly emarginate in middle of posterior margin, the middle emargination with lateral angles feebly protuberant and rounded at apices, behind it with a membranous lobe.

Internal reproductive organ (Fig. 4D): diverticulum moderately long, spermathecal duct thinner than and nearly as long as ventro-apical tube of vagina, spermatheca as long as diverticulum, accessory gland expanded at apical part, almost as long as spermatheca, the tube at base of spermatheca longer than spermathecal duct.

##### Diagnosis.

The new species is similar to *S. volaticus* in the shape of laterophyse of aedeagus (Fig. 6E vs. Wittmer 1974: fig. 9), but differs from the latter in the combination of the following characters: pronotum with anterior margin nearly straight, lateral margins sinuate (Fig. 5D), while anterior margin feebly arcuate, lateral margins rounded in the latter (Fig. 5A); elytra black (Fig. 5D), while dark brown in the latter (Fig. 5A); abdominal sternite VIII with a shallow and wide rectangular emargination in middle of posterior margin (Fig. 3D), while with a deeper and narrow rounded emargination in the latter (Fig. 3B; Švihla 2004: fig. 209); aedeagus with dorsal plates of parameres narrowly rounded at apical margins, with inner margins converging apically (Fig. 6F), while truncate at apical margins, with inner margins diverging apically in the latter (Wittmer 1974: fig. 9).

##### Etymology.

The species is named after the collector of the type material, Dr Hongbin Liang (IZAS).

##### Distribution.

(Fig. 1) China (Xizang).

## Supplementary Material

XML Treatment for Stenothemus benesi

XML Treatment for Stenothemus dundai

XML Treatment for Stenothemus jindrai

XML Treatment for Stenothemus jindraimimus

XML Treatment for Stenothemus shaanxiensis

XML Treatment for Stenothemus schneideri

XML Treatment for Stenothemus separatus

XML Treatment for Stenothemus ganeshai

XML Treatment for Stenothemus volaticomimus

XML Treatment for Stenothemus volaticus

XML Treatment for Stenothemus yunnanus

XML Treatment for Stenothemus dromoensis

XML Treatment for Stenothemus liangi

## Additional information

### Artificial Intelligence (AI) use

The authors accept full responsibility for the content of the manuscript, including the disclosure of any use of AI.

No AI tools were used in the preparation of this manuscript.

### Author ORCIDs

R. Hua https://orcid.org/0009-0007-3649-9105

S. Ge https://orcid.org/0000-0002-3621-5068

H. Liu https://orcid.org/0000-0003-1383-5560

X. Yang https://orcid.org/0000-0003-3676-6828

Y. Yang https://orcid.org/0000-0002-3118-6659

### Data availability

All of the data that support the findings of this study are available in the main text.
